# Sexual violence in the lives of first-year university women in Canada: no improvements in the 21st century

**DOI:** 10.1186/s12905-014-0135-4

**Published:** 2014-11-05

**Authors:** Charlene Y Senn, Misha Eliasziw, Paula C Barata, Wilfreda E Thurston, Ian R Newby-Clark, H Lorraine Radtke, Karen L Hobden, SARE Study Team

**Affiliations:** Department of Psychology/Women’s Studies Program, University of Windsor, 401 Sunset Avenue, Windsor, ON N9B 3P4 Canada; Department of Public Health and Community Medicine, Tufts University, 136 Harrison Avenue, Boston, MA 02111 USA; Department of Community Health Sciences, University of Calgary, 3280 Hospital Drive NW, Calgary, AB T2N 4Z6 Canada; Department of Psychology, University of Guelph, Guelph, ON N1G 2W1 Canada; Department of Ecosystem and Public Health, University of Calgary, 3280 Hospital Drive NW, Calgary, AB T2N 4Z6 Canada; Department of Psychology, University of Calgary, 2500 University Drive NW, Calgary, AB T2N 1 N4 Canada

**Keywords:** Sexual assault, Sexual coercion, Rape, Sexual violence, University women, Female university students

## Abstract

**Background:**

Summarizes the frequency, type, and context of sexual assault in a large sample of first-year university women at three Canadian universities.

**Methods:**

As part of a randomized controlled trial assessing the efficacy of a sexual assault resistance education program, baseline data were collected from women between ages of 17 and 24 using computerized surveys. Participants’ experience with sexual victimization since the age of 14 years was assessed using the Sexual Experiences Survey--Short Form Victimization (SES-SFV).

**Results:**

Among 899 first-year university women (mean age = 18.5 years), 58.7% (95% CI: 55.4%, 62.0%) had experienced one or more forms of victimization since the age of 14 years, 35.0% (95% CI: 31.9%, 38.3%) had experienced at least one completed or attempted rape, and 23.5% (95% CI: 20.7%, 26.4%) had been raped. Among the 211 rape victims, 46.4% (95% CI: 39.7%, 53.2%) had experienced more than one type of assault (oral, vaginal, anal) in a single incident or across multiple incidents. More than three-quarters (79.6%; 95% CI: 74.2%, 85.1%) of the rapes occurred while women were incapacitated by alcohol or drugs. One-third (33.3%) of women had previous self-defence training, but few (4.0%) had previous sexual assault education.

**Conclusions:**

Findings from the first large Canadian study of university women since the 1990s indicate that a large proportion of women arrive on campuses with histories of sexual victimization, and they are generally unprepared for the perpetrators they may face during their academic years. There is an urgent need for effective rape prevention programs on university campuses.

**Trial registration:**

ClinicalTrials.gov NCT01338428. Registered 13 April 2011.

## Background

Research suggests that the risk of sexual assault is particularly high for university women especially in the first year or two, which some authors have termed the ‘red zone’ [1,2]. In a representative sample of female students at Canadian universities, Dekeseredy and Kelly [3] found that more than one in four women have been sexually assaulted. Similar findings have been published in the U.S. [4,5]. This is particularly concerning given the detrimental physical and mental health consequences resulting from sexual assault, which include physical injuries as well as unwanted pregnancies, sexually transmitted diseases, depression, suicide ideation, and posttraumatic stress disorder [6,7]. Although university campuses in the U.S. have been identified as key sites for sexual assault prevention for more than 20 years [8], renewed 2005 and 2013], prevention efforts have had limited effects. President Obama recently called for an urgent and systematic approach to address the issue of sexual violence on campus [9]. There are no similar directives or federal or provincial regulations for campuses in Canada.

Obama’s task force report, and others before it [10], stress that medical and psychological staff in campus health centres must be involved in dealing with sexual violence. Because most women do not seek out or disclose to formal health resources [11], appropriate and accessible intervention may be the key to minimizing these health effects. As increased support and prevention efforts take shape in Canada [12,13], it is essential that we understand the types of sexual violence, and their prevalence, that first-year university women experience to better tailor victim support, health care, and prevention.

Although it is clear that rates of sexual assault in the general Canadian population have not decreased over the past 20 years [14,15], there have been few studies of sexual violence on Canadian campuses since the early 1990s. With one exception [16], the studies were based on small samples and at single universities. The aim of the present study is to rectify these deficiencies by providing data on the frequency, type, and context of sexual assault experiences in a large sample of women attending their first year of university in three Canadian cities. This article summarizes women’s experiences with sexual coercion and sexual assault since the age of 14 years, provides a brief sketch of their voluntary sexual and romantic relationship history, and their prior education on sexual assault and self-defence. These results should prove useful to university administrators, physicians, health centre and student services staff and student groups in planning and implementing prevention and treatment efforts.

## Methods

### Participants

Eligible participants were 1^st^ year university women, 17 to 24 years of age, and enrolled between 2011 and 2013 at three Canadian universities (one large university in Western Canada, two midsized universities in central Canada). The data for the present study were gathered within the context of a randomized controlled trial evaluating a sexual assault resistance education program for university women [17]. Ethics approval for the trial was received from the University of Windsor Research Ethics Board, from the University of Guelph’s Research Ethics Board, and from the University of Calgary’s Conjoint Health Research Ethics Board.

### Enrolment procedures

Students who met the eligibility criteria were recruited in the fall and winter semesters for two consecutive years through a variety of methods. Most (63.0%) participants were recruited through emails and phone calls to first-year female students registered in psychology research systems. The remaining participants were recruited through other means (e.g., in large, first-year courses, through professors or friends, through posters or flyers around campus; during orientation and other student events). The study was described in its briefest form as “an evaluation of campus sexual assault education interventions for university women”.

All interested women had direct communication with a research assistant who explained the full trial protocol [17] before they registered for the baseline survey. Upon arrival at a computer laboratory, participants were greeted by a research assistant, signed a consent form, and completed a computerized survey using MediaLab [18], (seated so they could not easily see the screens of other participants). After completing the survey, participants were randomized and sent to their assigned intervention session. Incentives for survey completion consisted of either a ballot towards a $300 lottery or bonus points towards their psychology course grade. All participants were provided with a list of in-person and online campus and community advocacy and counselling resources. Research assistants were available to provide temporary support and referrals to resources should participants show any signs of upset.

### Measures

Among other items, the survey consisted of (in order) demographics (age, ethnicity, sexual identity, and living arrangements), relationship history, perceived risk questions, sexual violence victimization, and exposure to sexual assault education and self-defence, including recency and duration of sessions. Relationship history included age at first sexual partner, number of previous sexual partners, whether ever had a relationship with a man, and whether or not currently in a romantic and/or sexual relationship (‘sexual’ and ‘sexual partner’ were self-defined).

Perception of risk of sexual assault by male acquaintances was measured by two items adapted from [19] “What are the chances of a woman your age being raped by someone she knows?” and “What are your chances of being raped by someone you know?”

Sexual violence victimization information was collected using the *Sexual Experiences Survey -- Short Form Victimization* (SES-SFV) [20]. The SES-SFV is a revision of the original Sexual Experiences Survey (SES) developed in 1982 [21,22] which is the most widely used scale for gathering data about sexual violence [20]. A strength of the SES-SFV is that the questions provide behavioural descriptions that describe what is legally considered to be sexual assault (in Canada) and rape (in United States) without the corresponding labels [20]. A gender-specific version of the wording was used (i.e., measured male perpetration only).

The SES-SFV provides participants with seven items consisting of a stem describing a sexually coercive or assaultive act or attempted act (e.g., “A man put his penis into my vagina, or inserted fingers or objects without my consent by:”) followed by five descriptions of perpetrator tactics or strategies (e.g., “Using force, for example holding me down with their body weight, pinning my arms, or having a weapon”). The tactics range from verbal pressure to physical force. Participants respond to each stem and tactic based on frequency of occurrence (0, 1, 2, 3 or more times) since age 14 years. Standard coding creates six categories of victimization based on severity: non-victim, sexual contact, attempted coercion, coercion, attempted rape, and rape. Because participants are limited to reporting each item to a maximum of 3 or more times (response option ‘3 or more’), the frequency of sexual violence is, somewhat, underestimated by the SES-SFV.

### Statistical analysis

Means and proportions were used to summarize the results, including 95% confidence intervals. T-tests and Chi-square tests were used to compare differences between group means and proportions, respectively. P-values less than 0.05 were considered to be statistically significant.

## Results

### Participant demographics

The mean age of the 899 eligible participants was 18.5 years (SD = 1.2 years). Although they were mostly (72.9%) White (of Canadian, Nordic or European heritage), a sizeable proportion were: Aboriginal (2.3%) or women of colour (24.1% self-identifying as Asian, South Asian, African, Latin/Central American, Filipino, Middle Eastern or bi-racial); and 0.7% did not disclose. Approximately one-half (54.1%) of the participants lived in a university residence, while the rest lived off-campus.

### Romantic, sexual and relationship history

Most (91.7%) women identified themselves as heterosexual and the majority (84.4%) reported a dating relationship with a man in the past. A sizeable minority (38.2%) reported they had never had a sexual partner. For the other 61.8% who had been sexually active, the mean age of their first sexual partner was 16.4 years (SD = 1.5 years) with the youngest being 12 years and the oldest 21. Less than half of all participants were currently involved in a romantic (44.5%) or sexual (45.0%) relationship (with anyone, male or female).

### Victimization and sexual assault since age 14 years

More than half (58.7%) had experienced one or more forms of victimization since the age of 14 years (Table 1), 35.0% had experienced at least one completed or attempted rape, and 23.5% had been raped. Sexual contact without penetration through the use of force, threats, or drugs, had been experienced by 50.5% of the women. Coercion of any type occurred frequently. Among the women who reported being victimized, the average number of occurrences was 4.8. Although not statistically significant, women who were recruited later in the academic year (winter semester) were more likely to have had experienced rape and attempted rape than women who were recruited earlier in the fall semester (rape: 25.3% versus 22.2%, p-value = 0.28; attempted rape: 30.0% versus 25.4%, p-value = 0.13).

**Table 1:** **Frequency (Percentage) and average number of victimizations since age 14 years among 899 first-year university women**

**Form of victimization**	Frequency (%*)	**95% CI**	**Average number of victimizations** ^**†**^	**95% CI**
Any form of victimization	528 (58.7)	(55.4, 62.0)	4.8	(4.4, 5.3)
Completed or attempted rape	315 (35.0)	(31.9, 38.3)	3.0	(2.7, 3.3)
Completed rape	211 (23.5)	(20.7, 26.4)	2.2	(2.0, 2.5)
Attempted rape	246 (27.4)	(24.5, 30.4)	2.4	(2.2, 2.7)
Coercion	200 (22.2)	(19.6, 25.1)	2.5	(2.2, 2.8)
Attempted coercion	274 (30.5)	(27.5, 33.6)	2.7	(2.5, 3.0)
Sexual contact	454 (50.5)	(47.2, 53.8)	2.3	(2.1, 2.4)

CI = Confidence interval.

*Percentages do not add to 100 because women may have had multiple forms of victimization.

^†^Geometric mean among women who were victimized one or more times since the age of 14 years; sample size is the frequency in the second column.

Nearly half of completed rape and attempted rape victims (46.4% and 50.8%) had experienced more than one type of assault in a single incident or across multiple incidents since the age of 14 years (Table 2). Although anal assaults were the least common, they were almost always in combination with other forms of rape and were experienced by approximately one in six victims. More than three-quarters of completed and attempted rapes (79.6% and 78.9%) occurred with incapacitation by alcohol or drugs in combination with other perpetrator tactics, and over one-half (50.7% and 54.1%) with alcohol or drugs alone (Table 3). However, men also used force in almost half (48.3% and 44.3%) of all completed and attempted rapes. Men’s use of threats was less common.

**Table 2:** **Types of sexual assaults experienced since age 14 years among women who reported completed rape (n = 211) and attempted rape (n = 246)**

**Type**	**Frequency (%)**	**95% CI**	**Any type**	**Frequency (%** ^**†**^ **)**	**95% CI**
**Completed rape**					
Oral only	44 (20.8)	(15.4, 26.3)	Any oral	133 (63.0)	(56.5, 69.5)
Vaginal only	68 (32.2)	(25.9, 38.5)	Any vaginal	162 (76.8)	(71.1, 82.5)
Oral and vaginal*	65 (30.8)	(24.6, 37.0)	Any anal	34 (16.1)	(11.1, 21.1)
Anal only	1 (0.5)	(0.0, 2.6)	More than one type	98 (46.4)	(39.7, 53.2)
Anal and oral*	4 (1.9)	(0.5, 4.8)			
Anal and vaginal*	9 (4.3)	(2.0, 8.0)			
Anal, vaginal, and oral*	20 (9.5)	(5.5, 13.4)			
**Attempted rape**					
Oral only	60 (24.4)	(19.0, 29.8)	Any oral	182 (74.0)	(68.5, 79.5)
Vaginal only	57 (23.2)	(17.9, 28.4)	Any vaginal	179 (72.8)	(67.2, 78.3)
Oral and vaginal*	94 (38.2)	(32.1, 44.3)	Any anal	35 (14.2)	(9.9, 18.6)
Anal only	4 (1.6)	(0.4, 4.1)	More than one type	125 (50.8)	(44.6, 57.1)
Anal and oral*	3 (1.2)	(0.2, 3.5)			
Anal and vaginal*	3 (1.2)	(0.2, 3.5)			
Anal, vaginal, and oral*	25 (10.2)	(6.4, 13.9)			

CI = Confidence interval.

*The category may represent more than one type of assault in a single incident or across multiple incidents.

^†^Percentages do not add to 100 because women may have had multiple types.

**Table 3:** **Types of perpetrator tactics used for women who reported completed rape (n = 211) and attempted rape (n = 246) since age 14 years**

**Type**	**Frequency (%)**	**95% CI**	**Any type**	**Frequency (%** ^**†**^ **)**	**95% CI**
**Completed rape**					
Alcohol/drug only	107 (50.7)	(44.0, 57.5)	Any alcohol/drug	168 (79.6)	(74.2, 85.1)
Force only	37 (17.5)	(12.4, 22.7)	Any force	102 (48.3)	(41.6, 55.1)
Alcohol/drug and force*	50 (23.7)	(18.0, 29.4)	Any threats	17 (8.1)	(4.4, 11.7)
Threats only	1 (0.5)	(0.0, 2.6)	More than one type	66 (31.3)	(25.0, 37.5)
Alcohol/drug and threats*	1 (0.5)	(0.0, 2.6)			
Force and threats*	5 (2.4)	(0.8, 5.4)			
Alcohol/drug, force, and threats*	10 (4.8)	(2.3, 8.5)			
**Attempted rape**					
Alcohol/drug only	133 (54.1)	(47.8, 60.3)	Any alcohol/drug	194 (78.9)	(73.8, 84.0)
Force only	44 (17.9)	(13.1, 22.7)	Any force	109 (44.3)	(38.1, 50.5)
Alcohol/drug and force*	49 (19.9)	(14.9, 24.9)	Any threats	20 (8.1)	(4.7, 11.5)
Threats only	2 (0.8)	(0.1, 2.9)	More than one type	67 (27.2)	(21.7, 32.8)
Alcohol/drug and threats*	2 (0.8)	(0.1, 2.9)			
Force and threats*	6 (2.4)	(0.9, 5.2)			
Alcohol/drug, force, and threats*	10 (4.1)	(2.0, 7.3)			

CI = Confidence interval.

*The category may represent more than one type of tactic in a single incident or across multiple incidents. ^†^Percentages do not add to 100 because women may have had multiple types.

### Prior sexual assault education and self-defence training

Few (4.0%) first-year university women had ever taken an “in-depth workshop or training on sexual assault or rape”. Among the 36 women who had, approximately one-half (55.6%) of them took the training more than two years earlier and for one-third (33.3%) the training was three hours or less in length. From responses to the open-ended question, it was clear that some of these experiences were self-defence training that mentioned sexual assault. In contrast, one-third (33.3%) of women reported previous self-defence training. Among the 299 women, 81.6% reported having training more than two years earlier. Approximately two-thirds (65.9%) had brief training (i.e., less than one-half day, most less than 3 hours), but a sizeable proportion (34.1%) reported more extensive self-defence training of a full day or more.

### Perception of risk of sexual assault by an acquaintance

Participants differed markedly in their perceptions of other women’s risk of acquaintance rape versus their own. On the five-point scale ranging from ‘very unlikely’ to ‘very likely’ , over one-half (56.6%) believed that the chances were either ‘likely’ or ‘very likely’ that other women their age could be raped by an acquaintance, while only a small number (6.9%) thought that the chances of acquaintance rape were either ‘likely’ or ‘very likely’ for themselves. The 211 women who were previously raped perceived their own (14.7%) and other women’s risk (66.3%) of being raped as significantly higher than did the 688 women who had never been raped (4.5% and 53.6%, respectively).

### Effect of recruitment strategy on demographic and sexual assault variables

The possibility of differential selection bias affecting the characteristics of the sample was examined by comparing the 566 women recruited through the psychology research systems to the 333 women recruited through other methods (Table 4). There were no significant differences between the two groups, except that women recruited through the psychology research systems were less likely to live in a university residence and were less likely to have been victimized or have had (forced, threatened or drugged) sexual contact.

**Table 4:** **Comparison of participant characteristics and victimizations by method of recruitment**

	**Research systems (N = 566)**	**Other methods (N = 333)**	**P-value**
Age in years (mean)	18.5	18.4	0.34
White race (%)	73.0	72.7	0.92
Living in a university residence (%)	45.4	68.8	< 0.001
Heterosexual (%)	93.1	89.2	0.04
Sexually active (%)	61.7	62.2	0.88
Age in years of first sexual partner (mean)	16.4	16.5	0.49
Currently involved in a romantic relationship (%)	44.9	43.8	0.76
Currently involved in a sexual relationship (%)	43.3	48.0	0.17
Prior sexual assault education (%)	3.7	4.5	0.56
Prior self-defence training (%)	31.8	35.7	0.23
Perceived personal risk of sexual assault as either ‘likely’ or ‘very likely’ (%)	6.7	7.2	0.78
Any form of victimization (%)	56.0	63.4	0.031
Completed rape (%)	21.9	26.1	0.15
Sexual contact (%)	47.0	56.5	0.006
More than one type of sexual assault during completed rape (%)	50.0	41.4	0.22
More than one type of perpetrator tactic during completed rape (%)	33.9	27.6	0.33

## Discussion

The present study is the first large, university-based sexual assault study in Canada since the 1990s [3]. A majority of first-year female students had been sexually assaulted by a man, with one-third previously having experienced rape or attempted rape. Incapacitation due to drugs or alcohol and use of force were the most common perpetrator tactics. The results are suggestive of a ‘red zone’ [2], whereby the occurrence of sexual victimization was higher among women who were recruited later in the academic year (winter semester) than earlier (fall semester). A comparison to the best U.S. studies conducted within the last 15 years confirm these findings [5]. Re-victimization was found to occur commonly in the present study and elsewhere [23], and it has been shown to have a greater negative impact on victims [24], and future victimization may be more difficult to prevent.

The study results also suggest that only a small minority of women have previous education or training about sexual assault, and therefore most are unprepared for the perpetrators they may face once they arrive on campus. Although high quality self-defence training has been shown to be empowering for women [25], because so few participants reported they also received information about rape, it seems likely this was ‘stranger danger’ self-defence. Without debunking rape myths, such training is unlikely to be put into practice by women against men they know [26]. In addition, the optimism bias held by these first-year students (and women more generally [27]) that other women are at greater risk of sexual assault than they are suggests that young women are not well prepared. This pattern was also evident, although less pronounced, for women who had previously been raped by men.

The present collection of findings has a number of implications for policy and prevention efforts on campus, with women and with men. First, it is well-known that alcohol and drugs are implicated in many of the sexual assaults of women students in university [28]. However, the relationship between alcohol and sexual assault is not simple nor direct [29]. Although decreasing alcohol consumption among male and female students is likely to reduce sexual violence perpetration, the present results do not support the premise that alcohol-involved assaults are the result of miscommunication between students who have simply had too much to drink or that sexual assault would end if women drank less, as commonly emphasized by the media and university programming. Our results show that men used force or threats in more than half of the sexual assaults committed, including situations where women were also incapacitated (voluntarily or involuntarily) by drugs or alcohol. More productive interventions for women should focus on safety planning and resistance across varied social situations, including ones involving alcohol or drugs, since women’s behavioral reactions to threat may be slowed or reduced when alcohol is consumed [30].

Second, the present study supports findings from U.S. studies showing that many young women will have experienced sexual assault before they turn 18 years of age [31] or by the time they enter university. Staff conducting education programming must be aware that women who have experienced rape or other forms of sexual assault will be present in student audiences. Several ‘risk reduction’ programs have been differentially effective for women with and without a history of sexual assault, showing no effects for previously victimized women [32,33]. In particular, program content that could be interpreted by a survivor as victim-blaming could be harmful and potentially increase detrimental health outcomes. Efficacious education and prevention efforts need to be expanded that take abuse history into account, and begin as soon as possible when a woman first enters university [1]. In addition, provision of student support and services should be trauma-informed [34,35], with all front-line staff receiving appropriate training. Without these measures, sexual assault rates can only be expected to increase by graduation.

Third, optimism biases regarding negative events are commonplace and sexual victimization is no exception. These biases may be obstacles for attracting female students to interventions or keeping them engaged. Interventions need to take a multi-prong approach to counteract optimism bias. Perceptions of personal relevance can be increased by using recent and local statistics [19]. Focusing on the development of situational risk detection skills that do not require personal identification with risk is also recommended [30].

Finally, while resistance interventions targeting women are one piece of the sexual violence prevention puzzle [36], effective interventions that can reach young men and create male allies are critical. Reducing men’s perpetration would be the most direct means to reduce sexual violence on campuses. To date, programs targeting men to attempt to prevent perpetration have been largely unsuccessful [37,38]. Bystander-type interventions which aim to create a community of men (and women) who recognize the cultural supports for sexual violence, object to demonstrations of sexually aggressive masculinity, and intervene in situations where sexual assault risk is apparent, show considerable promise e.g., [39]. Until these other programming efforts demonstrate reductions in sexual assault rates and widespread dissemination, resistance programming for women will be necessary.

A major strength of the present study is the use of the revised SES-SFV, which makes it possible for the first time to provide Canadian data on the frequency of oral, vaginal, and anal rape and attempted rape. Although oral and vaginal rapes were most common, anal rape, with its additional health risks, was experienced by one in six rape survivors and almost always in combination with other types of rape. Little attention has been paid to anal rape on university campuses, although a recent U.S. study suggests that it is a concern [40]. Anal rape is unlikely to be discussed in high school because explicit sexual content is often removed from sexual education programs when they are adapted for adolescents [41].

One limitation of the current study is that, due to the requirements in conducting a randomized controlled trial, we did not have a random sample of first-year university women. Participants had to be willing to participate in a sexual assault intervention. Based on prior research regarding subject selection biases [42], the manner in which our study was advertised could have enrolled a greater number of previously victimized and sexually experienced women than the general population of first-year women. Nevertheless, our large-sample study substantiates the need to address sexual assault risk as soon as possible after women enter university.

## Conclusions

The results of the present study provide a profile of women who were willing to participate in sexual assault resistance education and would, therefore, be receptive to it. As such, the study findings should be of particular interest to campus health centres, student services and university administrators. The continuing high rates of sexual assault indicate the need for effective services and interventions on campuses, including a requirement for primary care physicians and nurses to ask about sexual violence when taking patient histories [43]. Although there are challenges to prevention because many interventions lack theoretical grounding, empirical evaluation, or are generally ineffective or worse [44], another 20 years cannot be permitted to pass without a decline in sexual assault among women entering university. Fortunately, sexual assault resistance education for women is being developed [45-47], one program is currently being evaluated in a current randomized controlled trial [17], and other promising efforts to engage male and female bystanders in sexual assault prevention are underway [39,48].
